# A Cancer Research Campaign (CRC) phase II trial of CB10-277 given by 24 hour infusion for malignant melanoma.

**DOI:** 10.1038/bjc.1994.395

**Published:** 1994-10

**Authors:** N. M. Bleehen, A. H. Calvert, S. M. Lee, P. Harper, S. B. Kaye, I. Judson, M. Brampton

**Affiliations:** University Department, Addenbrooke's Hospital, Cambridge, UK.

## Abstract

The decarbazine analogue CB10-277 has been investigated for anti-tumour activity in a phase II study on malignant melanoma. Treatment was administered as a slow infusion of 12,000 mg m-2 over 24 h and repeated every 3 weeks. A total of 28 patients were entered into the study, of whom 23 were eligible for review. A total of 64 courses was given. There was one objective partial response in 22 patients assessable for response. The major toxicities were leucopenia and thrombocytopenia. CB10-277 in this schedule therefore does not appear to have major activity in melanoma.


Br. J. Cancer (1994), 70, 775-777 © Macmillan Press Ltd., 1994

### A Cancer Research Campaign (CRC) phase II trial of CB10-277 given by 24 hour infusion for malignant melanoma

N.M. Bleehen[1], A.H. Calvert[2], S.M. Lee[3], P. Harper[4], S.B. Kaye[5], I. Judson[6] & M. Brampton[7]

[1]University Department and MRC Unit of Clinical Oncology & Radiotherapeutics, Addenbrooke's Hospital, Cambridge CB2 2QQ, UK; [2]Division of Oncology, Cancer Research Unit, University of Newcastle upon Tyne, Newcastle upon Tyne NE2 4HH, UK; [3]Christie Hospital, Department of Medical Oncology, Wilmslow Road, Manchester M20 9BX, UK; [4]Guy's Hospital, Department of Medical Oncology, St Thomas Street, London SE1 9RT, UK; [5]The Beatson Oncology Centre, Western Infirmary, Glasgow G11 6NT, UK; [6]The Institute of Cancer Research, 15 Cotswold Road, Sutton SM2 5NG, UK; [7]CRC Data Centre, Department of Medical Oncology, Charing Cross Hospital, London W6 8RF, UK.

**Summary** The dacarbazine analogue CB10-277 has been investigated for anti-tumour activity in a phase II study on malignant melanoma. Treatment was administered as a slow infusion of 12,000 mg $m^{-2}$ over 24 h and repeated every 3 weeks. A total of 28 patients were entered into the study, of whom 23 were eligible for review. A total of 64 courses was given. There was one objective partial response in 22 patients assessable for response. The major toxicities were leucopenia and thrombocytopenia. CB10-277 in this schedule therefore does not appear to have major activity in melanoma.

The anti-tumour activity of triazenes was first reported in animals in 1962 (Shealy *et al.*, 1962). Dimethyl triazanoimidazole carboxamide (dacarbazine, DTIC) the lead compound in clinical practice, has been demonstrated to have activity in patients with lymphomas, melanomas and sarcomas (Beretta *et al.*, 1976). *In vivo* metabolic activation of dacarbazine by *N*-demethylation is thought to be required for anti-tumour activity (reviewed in Newell *et al.*, 1987) but may not be the sole determinant of cytotoxicity. In addition to metabolic activation, dacarbazine undergoes chemical decomposition which is light catalysed. Protection of dacarbazine solutions from light has been claimed to result in reduced systemic toxicity (Baird & Willoughby, 1978; Koriech & Shükla, 1981), and this is the current clinical practice. Furthermore, murine studies suggest that the occasional skin reactions seen in patients treated with dacarbazine, particularly at high doses, may have a photochemical basis (Dorr *et al.*, 1987). Photochemical decomposition is not thought to contribute to the anti-tumour activity of dacarbazine (Newell *et al.*, 1987; Julliard & Vernin, 1981), and therefore a photostable analogue of dacarbazine may have advantages over the parent drug.

The dacarbazine analogue 1-(4-carboxyphenyl)-3,3-dimethyltriazene, (CB10-277) is soluble and stable in aqueous solution at physiological pH (Wilman & Goddard, 1980). It requires activation, similar to dacarbazine, for its anti-tumour activity (Connors *et al.*, 1976) but may be more readily activated in rats (Rutty *et al.*, 1986). These structural similarities, with better stability in solution and possible improved metabolic activation, led to preclinical and phase I studies with the drug (Foster *et al.*, 1993*a*, *b*). In human melanoma xenografts and transplantable rodent tumours, CB10-277 showed a spectrum of activity similar to dacarbazine (Foster *et al.*, 1993*a*). In man, the dose-limiting toxicity was nausea and vomiting when CB10-277 was given by short-term infusion (up to 35 min) every 3 weeks. The maximum tolerated dose was 6,000 mg $m^{-2}$. Tumour responses were reported in patients with melanoma (one complete, two partial, out of 11 treated) as well as in one patient with recurrent carcinoid. Mixed responses were seen in one further melanoma and one sarcoma patient.

A second phase I trial modelled on the plasma pharmacokinetic studies in the previous study attempted to reduce the degree of nausea and vomiting by using a 24 h infusion schedule (Foster *et al.*, 1993*b*). It was believed that a decrease in peak plasma levels of parent CB10-277 might permit a larger total drug administration over 24 h with increased levels of the biologically active monomethyl metabolite. A recommended dose of 12,000 mg $m^{-2}$ was determined, with acceptable nausea and vomiting and minimal myelosupression. No tumour responses were recorded in this second study.

On the basis of the known activity of dacarbazine against melanoma, the 3/11 responses seen with short-term infusion of CB10-277 and the improved acceptability of the prolonged infusion the 24 h schedule was investigated in a CRC phase II study. The clinical results of that study are presented.

### Patients and methods

### Patients

Eligibility criteria required histologically proven malignant melanoma with measurable disease not amenable to locoregional treatment and documented to have progressed within the 2 months prior to entry into the study. Other criteria included performance status 0-2 (WHO scale), age 75 or less, white blood cell count (WBC) $3 \times 10^9 l^{-1}$ or higher, platelets $100 \times 10^9 l^{-1}$ or higher, serum bilirubin below 20 $\mu$mol $l^{-1}$ and creatinine below 150 $\mu$mol $l^{-1}$. No prior chemotherapy, other than a biological response modifier given more than 4 weeks prior to study, was permitted, nor was prior radiotherapy to the measurable or evaluable disease. Patients with poor general medical risk were excluded.

Before receiving the first course of CB10-277 baseline investigations included physical examination, chest radiography and other appropriate radiological studies to document the extent of the disease. Blood chemistry (urea, creatinine, uric acid, electrolytes, calcium, glucose, bilirubin, alkaline phosphatase and ALT) and full blood counts (haemoglobin, white cell and differential, platelets) were recorded before treatment and weekly during treatment. Clinical assessment of evaluable lesions was documented before each course and on termination of therapy. Evaluation for side effects and response followed strict WHO criteria (WHO, 1979). In patients with more than one evaluable lesion the least responsive indicator lesion determined the overall response assessment.

The study was approved by the Medical Ethics Committees of the participating centres and informed consent was obtained from each patient.

Correspondence: N.M. Bleehen.
Received 29 April 1994; and in revised form 14 June 1994.

*Drug*

CB10-277 (MW = 215) was formulated as the sodium salt as a lyophilised pyrogen- and preservative-free powder in 1,000 mg vials by the Developmental Therapeutics Program, National Cancer Institute, Bethesda, MD, USA.

For each course the patient's total dose was calculated and then half was reconstituted (50 mg $ml^{-1}$) and placed in a 1,000 ml 0.9% normal saline bag and infused over 12 h. The remaining half of the dose was then reconstituted and given over the second 12 h. The infusion was carried out in this manner because the drug preparation contained no bacteriostatic agent.

*Treatment schedule*

A dose of 12,000 mg $m^{-2}$ was selected on the basis of the phase I study given as a slow infusion of 24 h (Foster *et al.*, 1993*b*).

All patients were given standard antiemetics including metoclopramide, chlorpromazine, lorazepam and recently ondansetron.

Provided that there was no disease progression, courses were repeated every 21 days. Dose modification was indicated on the basis of haematopoietic toxicity with reductions by 25% for WBC $<2 \times 10^9 l^{-1}$ and platelets $<75 \times 10^9 l^{-1}$ on the day of the next treatment or of 50% if morbidity for infection or haemorrhage. Dose delays were at the discretion of the individual clinicians.

Data collection and initial analysis were carried out by the CRC Phase I/II Trials Office and subsequently reviewed by the two study chairmen.

### Results

A total of 28 patients was entered into the study, of whom 23 were eligible for review. The five exclusions were due to an incorrect histological diagnosis (1), prior chemotherapy (2) and a raised serum bilirubin on entry (2). Patient characteristics are shown in Table I. One patient refused to continue in the study after the first course and was not assessable for response but has been included in this report. All the remaining 22 patients were assessable for toxicity and response after at least one full course of the drug.

A total of 64 courses of the drug were given with a median of 2 and a range of 1–6. There was a dose reduction in six courses in four patients (neutropenic septicaemia, 3; severe leucopenia and thrombocytopenia, 1; other, 2) to 50–75% of the planned dose. Treatment was delayed on 11 occasions (nine patients) owing to bed unavailability (2), surgical intervention (2) and haematological toxicity (5).

There was one objective partial response in a patient with extensive lymph node disease (duration 18 weeks) in the 22 patients assessable for response. Five patients had static disease over 1–6 courses (median two courses) before disease progression. The remaining patients had progressive disease.

**Table I** Patient characteristics

| | |
|---|---|
| Total number of patients entered | 28 |
| Total number of eligible patients[a] | 23 |
| Sex: male/female | 12/11 |
| Age median range (years) | 46 (20–70) |
| WHO performance status | |
| 0 | 5 |
| 1 | 17 |
| 2 | 1 |
| Prior treatment | |
| Surgery | 23 |
| Radiotherapy | 1 |
| None | 0 |
| Sites of assessable disease | |
| Primary/skin | 8 |
| Lymph nodes | 13 |
| Lung | 3 |
| Liver | 4 |
| Bone | 1 |
| Other | 3 |
| Total courses in 23 patients | 64 |
| Median courses (range) | 2 (1–6) |

[a]Five exclusions because of incorrect diagnosis (1), prior chemotherapy (2), raised bilirubin on entry (2).

**Table II** Toxicity

| Toxicity type | WHO toxicity grade: 0 or unrelated to CB10-277 | 1 | 2 | 3 | 4 |
|---|---|---|---|---|---|
| Nausea/vomiting | 4 | 2 | 9 | 8 | – |
| Haemoglobin | 18 | – | 3 | 1 | 1 |
| WBC | 19 | – | 3 | 2 | 2 |
| Platelets | 18 | – | – | 3 | 2 |
| Alopecia | 21 | 2 | – | – | – |
| Constipation | 19 | 1 | 2 | 1 | – |
| Renal (raised urea, creatinine, haematuria) | 20 | 2 | 1 | – | – |
| Liver (raised ALT) | 21 | – | 2 | – | – |
| Diarrhoea | 20 | 2 | – | 1 | – |
| Oral | 20 | 1 | 2 | – | – |
| Consciousness | 19 | 3 (lethargy) | 1 | – | – |
| Infection | 20 | – | 1 | 2 | – |

Others: Mild flushing, 1 patient; malaise, 2; muffling in ears, 1; anorexia, 4; moderate shivering 1; tiredness, 1; insomnia, 1; moderate headache, 1; weakness, 1; sweating, 2.

*Toxicities*

The most frequent toxicities are listed in Table II. The major toxicities were leucopenia and thrombocytopenia. Septicaemia was seen in two patients. Anaemia was also reported, but only possibly related to the drug as it occurred during the first cycle of treatment and in most patients was reported by the clinician as probably unrelated. Nausea and vomiting was common but usually controlled when ondansetron with dexamethasone was given. Constipation reported by some patients was also difficult to assess and may have been related to the ondansetron and opiates also given, and was not reported in the phase I studies in which ondansetron was not used (Foster *et al.*, 1993*a*, *b*). Numerous other minor symptoms were variously reported during the study but were of dubious relationship to drug administration and likely to have been due to disease progression.

### Discussion

The results of this study are disappointing in that only one partial responder was seen in the 22 patients with melanoma who could be assessed following treatment with 12,000 mg $m^{-2}$ CB10-277 given by 24 h infusion every 21 days. This result is disappointing in view of the anti-tumour efficacy of its analogue dacarbazine and the three responses in 11 patients documented in the phase I study with CB10-277 given over a short-term infusion (Foster *et al.*, 1993*a*). In that phase I study, 8/11 patients had received prior chemotherapy with a dacarbazine-containing regimen, including one of the partial responders to the subsequent CB10-277. In this present study none of the 22 patients had received any prior chemotherapy. However, this difference may be due to chance within the limits of the number of patients entered.

Pharmacokinetics were not studied in this phase II trial but had been extensively investigated in the two phase I studies (Foster *et al.*, 1993*a*, *b*). On the basis of those studies it was believed that the 24 h infusion would permit higher total drug doses with increased exposure to the active monomethyl metabolite over that afforded by the short-term infusion. At the time that the phase I studies were performed, the $5HT_3$

receptor antagonists currently used as antiemetics were not generally available. Nausea and vomiting was determined as the dose-limiting toxicity and influenced the decision to use the 24 h schedule for this study. Subsequently, ondansetron (Glaxo) became available and was successfully used in most of the patients in this study. It might be speculated that in view of the higher response rate seen in the short-infusion phase I study, a phase II study using the maximum tolerated dose over 30 min might have elicited further responses, especially if the nausea and vomiting were controlled by a more modern antiemetic.

One additional problem with this study is that the $O^6$-methylguanine methyltransferase activity of the tumours was not routinely assessed. A major target of the monomethyl metabolite of CB10-277 is $O^6$ alkylation of the guanine residues in DNA (Gibson *et al.*, 1986). The majority of human tumours possess high activities of the enzyme ($Mer^+$) and are therefore capable of repairing the DNA alkylation. The limited efficacy seen in the current study suggests that many of the 22 patients assessed may have had $Mer^+$ tumours. Supporting this suggestion are data from studies with human melanoma xenografts where in three tumours response was related to the level of the $O^6$ methylguanine methyltransferase repair protein, the only sensitive tumour having very low levels (Foster *et al.*, 1990).

The toxicity of the regimen was acceptable, but the lack of tumour responses does not encourage its use in melanoma in this 24 h schedule. Furthermore, the recent identification of temozolomide as an oral monomethyl triazene prodrug (Newlands *et al.*, 1992) with activity against melanoma suggests that future studies should concentrate on this agent, rather than CB10-277.

### References

BAIRD, G.M. & WILLOUGHBY, M.L.N. (1978). Photodegradation of dacarbazine. *Lancet*, **23**, 681.

BERETTA, G., BONADONNA, G., BAJETTA, E., TANCINI, G., DE LENA, M., AZZARELLI, A. & UMBERTO, V. (1976). Combination chemotherapy with DTIC (NSC-45388) in advanced malignant melanoma, soft tissue sarcoma and Hodgkin's disease. *Cancer Treat. Rep.*, **60**, 205–211.

CONNORS, T.A., GODDARD, P.M., MERAI, K., ROSS, W.C.J. & WILMAN, D.E.V. (1976). Tumour inhibitory triazenes: structural requirements for an active metabolite. *Biochem. Pharmacol.*, **25**, 241–246.

DORR, R.T., ALBERTS, D.S., EINSPAHR, J., MASON-LIDDIL, N. & SOBLE, M. (1987). Experimental dacarbazine antitumor activity and skin toxicity in relation to light exposure and pharmacologic antidotes. *Cancer Treat. Rep.*, **71**, 267–272.

FOSTER, B.J., NEWELL, D.R., LUNN, J.M., JONES, M. & CALVERT, A.H. (1990). Correlation of dacarbazine and CB10-277 activity against human melanoma xenografts with $O^6$ alkyltransferase. *Proc. Am. Assoc. Cancer Res.*, **31**, 401.

FOSTER, B.J., NEWELL, D.R., CARMICHAEL, J., HARRIS, A.L., GUMBRELL, L.A., JONES, M., GOODARD, P.M. & CALVERT, A.H. (1993*a*). Preclinical, phase I and pharmacokinetic studies with the dimethyl phenyltriazene CB10-277. *Br. J. Cancer*, **67**, 362–368.

FOSTER, B.J., NEWELL, D.R., GUMBRELL, L.A., JENNS, K.E. & CALVERT, A.H. (1993*b*). Phase I trial with pharmacokinetics of CB10-277 given by 24 hours continuous infusion. *Br. J. Cancer*, **67**, 369–373.

GIBSON, N.W., HARTLEY, J., LA FRANCE, R.H. & VAUGHAN, K. (1986). Differential cytotoxicity and DNA-damaging effects produced in human cells of the Mer+ and Mer− phenotypes by a series of 1-aryl-3 alkyltriazenes. *Cancer Res.*, **46**, 4999–5003.

JULLIARD, M. & VERNIN, G. (1981). Biological properties of antitumour triazenes. *Ind. Eng. Chem. Prod. Res. Dev.*, **20**, 287–296.

KORIECH, O.M. & SHÜKLA, V.S. (1981). Dacarbazine (DTIC) in malignant melanoma: reduced toxicity with protection from light. *Clin. Radiol.*, **32**, 53–55.

NEWELL, D., GESCHER, A., HARLAND, S., ROSS, D. & RUTTY, C. (1987). N-methyl antitumour agents. *Cancer Chemother. Pharmacol.*, **19**, 91–102.

NEWLANDS, E.S., BLACKLEDGE, G.R.P., SLACK, J.A., RUSTIN, G.J.S., SMITH, D.B., STUART, N.S.A., QUARTERMAN, C.P., HOFFMAN, R., STENVES, M.F.G., BRAMPTON, M.H. & GIBSON, A.C. (1992). Phase I trial of temozolomide (CCRG 81045: M&B 39831: NSC 362856). *Br. J. Cancer*, **65**, 287–291.

RUTTY, C.J., GRAHAM, M.A., ABEL, G., JUDSON, I.R. & GODDARD, P.M. (1986). Preclinical evaluation of 1-p-carboxy-3,3-dimethylphenyltriazene (CB10-277) an alternative to DTIC. *Br. J. Cancer*, **54**, 194.

SHEALY, Y.F., MONTGOMERY, J.A. & LASTER Jr, W.R. (1962). Antitumor activity of triazenoimidazoles. *Biochem. Pharmacol.*, **11**, 674–676.

WHO (1979). *WHO Handbook for Reporting Results of Cancer Treatment*. WHO Offset Publication No. 48, pp. 14–25. WHO: Geneva.

WILMAN, D.E.V. & GODDARD, P.M. (1980). Tumour inhibitory triazenes, 2. Variation of antitumour activity within a homologous series. *J. Med. Chem.*, **23**, 1052–1054.


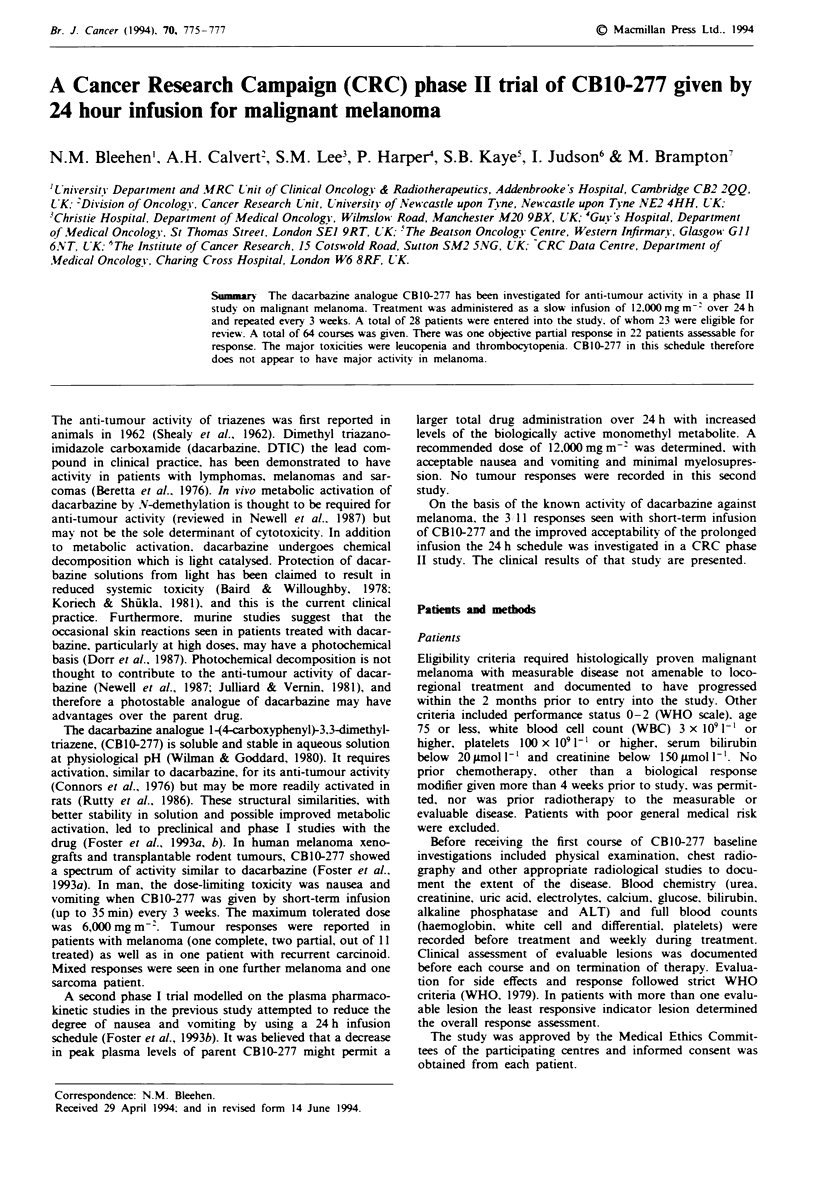

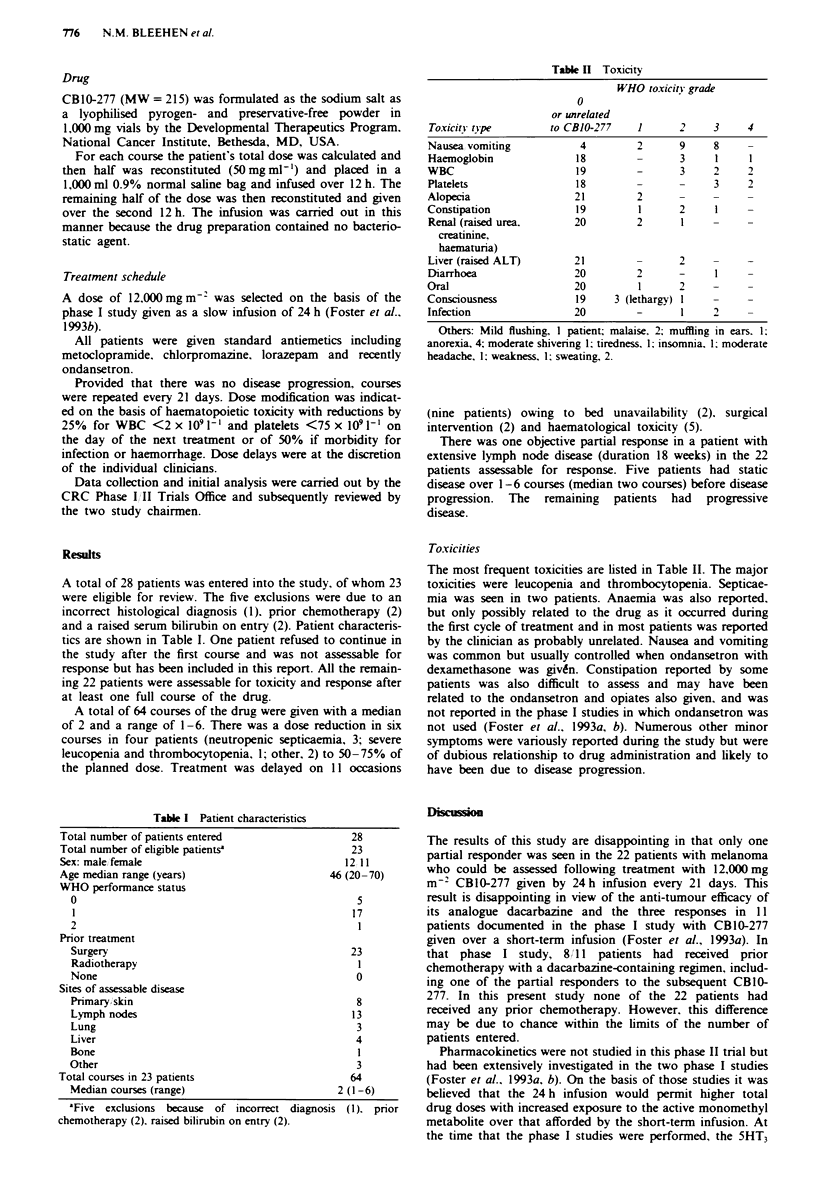

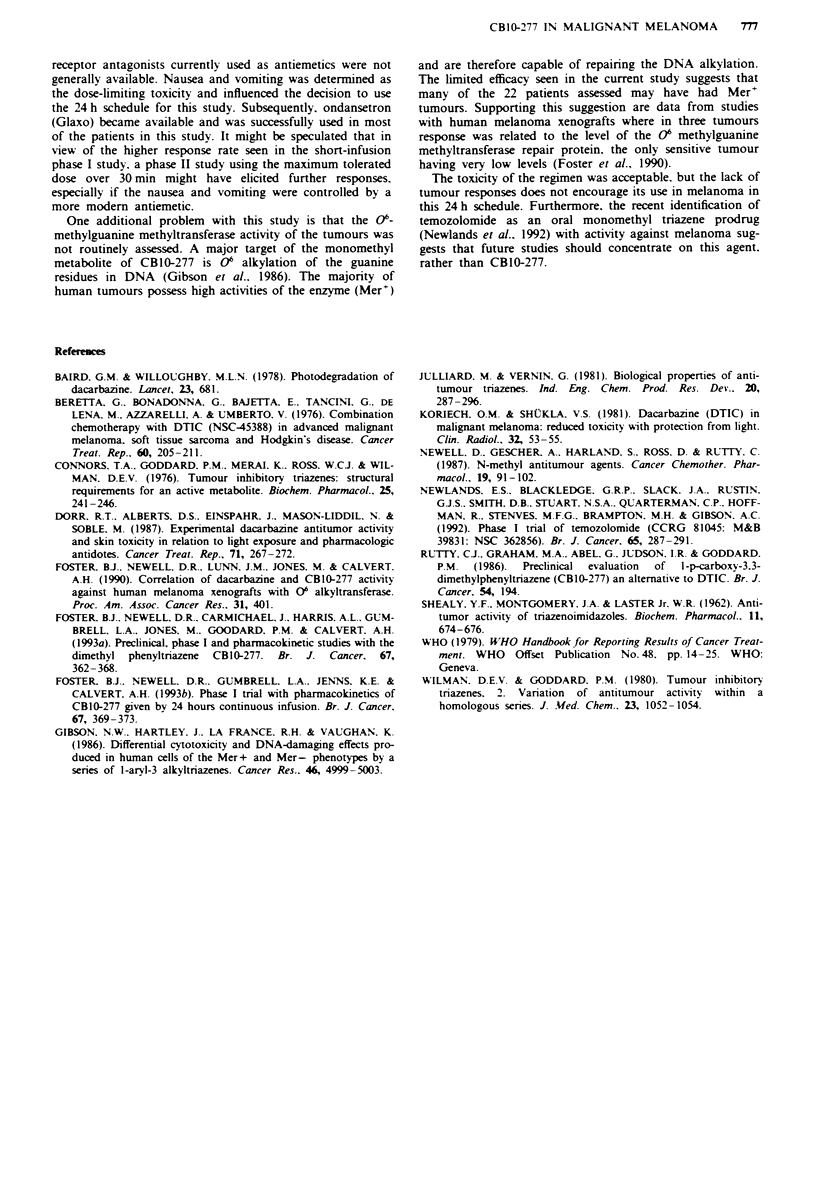


## References

[OCR_00357] Baird G. M., Willoughby M. L. (1978). Photodegradation of dacarbazine.. Lancet.

[OCR_00361] Beretta G., Bonadonna G., Bajetta E., Tancini G., De Lena M., Azzarelli A., Veronesi U. (1976). Combination chemotherapy with DTIC (NSC-45388) in advanced malignant melanoma, soft tissue sarcomas, and Hodgkin's disease.. Cancer Treat Rep.

[OCR_00368] Connors T. A., Goddard P. M., Merai K., Ross W. C., Wilman D. E. (1976). Tumour inhibitory triazenes: structural requirements for an active metabolite.. Biochem Pharmacol.

[OCR_00374] Dorr R. T., Alberts D. S., Einspahr J., Mason-Liddil N., Soble M. (1987). Experimental dacarbazine antitumor activity and skin toxicity in relation to light exposure and pharmacologic antidotes.. Cancer Treat Rep.

[OCR_00388] Foster B. J., Newell D. R., Carmichael J., Harris A. L., Gumbrell L. A., Jones M., Goodard P. M., Calvert A. H. (1993). Preclinical, phase I and pharmacokinetic studies with the dimethyl phenyltriazene CB10-277.. Br J Cancer.

[OCR_00395] Foster B. J., Newell D. R., Gumbrell L. A., Jenns K. E., Calvert A. H. (1993). Phase I trial with pharmacokinetics of CB10-277 given by 24 hours continuous infusion.. Br J Cancer.

[OCR_00399] Gibson N. W., Hartley J. A., LaFrance R. J., Vaughan K. (1986). Differential cytotoxicity and DNA-damaging effects produced in human cells of the Mer+ and Mer- phenotypes by a series of 1-aryl-3-alkyltriazenes.. Cancer Res.

[OCR_00410] Koriech O. M., Shükla V. S. (1981). Dacarbazine (DTIC) in malignant melanoma: reduced toxicity with protection from light.. Clin Radiol.

[OCR_00417] Newell D., Gescher A., Harland S., Ross D., Rutty C. (1987). N-methyl antitumour agents. A distinct class of anticancer drugs?. Cancer Chemother Pharmacol.

[OCR_00422] Newlands E. S., Blackledge G. R., Slack J. A., Rustin G. J., Smith D. B., Stuart N. S., Quarterman C. P., Hoffman R., Stevens M. F., Brampton M. H. (1992). Phase I trial of temozolomide (CCRG 81045: M&B 39831: NSC 362856).. Br J Cancer.

[OCR_00433] SHEALY Y. F., MONTGOMERY J. A., LASTER W. R. (1962). Antitumor activity of triazenoimidazoles.. Biochem Pharmacol.

[OCR_00443] Wilman D. E., Goddard P. M. (1980). Tumor inhibitory triazenes. 2. Variation of antitumor activity within an homologous series.. J Med Chem.

